# Pancreatic β-cell mitophagy as an adaptive response to metabolic stress and the underlying mechanism that involves lysosomal Ca^2+^ release

**DOI:** 10.1038/s12276-023-01055-4

**Published:** 2023-09-01

**Authors:** Soo-Jin Oh, Kihyoun Park, Seong Keun Sonn, Goo Taeg Oh, Myung-Shik Lee

**Affiliations:** 1 Soonchunhyang Institute of Medi-bio Science and Division of Endocrinology, Department of Internal Medicine, Soonchunhyang University College of Medicine, Cheonan, 31151 Korea; 2 Heart-Immune-Brain Network Research Center, Department of Life Science, Ewha Womans University, Seoul, 03767 Korea

**Keywords:** Metabolic syndrome, Macroautophagy

## Abstract

Mitophagy is an excellent example of selective autophagy that eliminates damaged or dysfunctional mitochondria, and it is crucial for the maintenance of mitochondrial integrity and function. The critical roles of autophagy in pancreatic β-cell structure and function have been clearly shown. Furthermore, morphological abnormalities and decreased function of mitochondria have been observed in autophagy-deficient β-cells, suggesting the importance of β-cell mitophagy. However, the role of authentic mitophagy in β-cell function has not been clearly demonstrated, as mice with pancreatic β-cell-specific disruption of *Parkin*, one of the most important players in mitophagy, did not exhibit apparent abnormalities in β-cell function or glucose homeostasis. Instead, the role of mitophagy in pancreatic β-cells has been investigated using β-cell-specific *Tfeb*-knockout mice (*Tfeb*^Δβ-cell^ mice); *Tfeb* is a master regulator of lysosomal biogenesis or autophagy gene expression and participates in mitophagy. *Tfeb*^Δβ-cell^ mice were unable to adaptively increase mitophagy or mitochondrial complex activity in response to high-fat diet (HFD)-induced metabolic stress. Consequently, *Tfeb*^Δβ-cell^ mice exhibited impaired β-cell responses and further exacerbated metabolic deterioration after HFD feeding. TFEB was activated by mitochondrial or metabolic stress-induced lysosomal Ca^2+^ release, which led to calcineurin activation and mitophagy. After lysosomal Ca^2+^ release, depleted lysosomal Ca^2+^ stores were replenished by ER Ca^2+^ through ER→lysosomal Ca^2+^ refilling, which supplemented the low lysosomal Ca^2+^ capacity. The importance of mitophagy in β-cell function was also demonstrated in mice that developed β-cell dysfunction and glucose intolerance after treatment with a calcineurin inhibitor that hampered TFEB activation and mitophagy.

## Diabetes: Quality control of pancreatic mitochondria is critical

A mechanism for eliminating damaged mitochondria in insulin-producing pancreatic beta cells provides an important safeguard against the onset of metabolic dysfunction and diabetes. Researchers have long suspected that this process, known as mitophagy, is essential to ß-cell survival and function, but past studies have yielded conflicting results. Myung-Shik Lee of Soonchunhyang University, Cheonan, South Korea, and colleagues have reviewed current knowledge about how mitophagy is regulated, focusing on a protein called TFEB, a master regulator of mitophagy. Rodent models in which TFEB activity is disrupted have provided compelling evidence that mitophagy plays a critical role in protecting beta cells. Pancreatic ß-cells from these animals are more susceptible to stress induced by a high-fat diet and show reduced capacity for glucose control, suggesting that mitophagy-modulating drugs may have therapeutic potential for diabetes.

## Introduction

Mitochondria play critical roles in the survival and function of pancreatic β-cells. ATP generated from glucose closes ATP-sensitive K^+^ channels, which induces membrane depolarization and consequent Ca^2+^ influx, accelerating insulin exocytosis^1^. In addition to insulin secretion, mitochondria play crucial roles in the survival or death of β-cells^2^. The maintenance of mitochondrial function relies on mitochondrial biogenesis, fission, and fusion, as well as the autophagic removal of mitochondria, which is termed mitophagy. Autophagy is an evolutionarily conserved and fundamental cellular process that facilitates the degradation and recycling of cellular components or organelles, such as mitochondria, to maintain cellular and organellar homeostasis. The crucial roles of autophagy in the maintenance of β-cell viability and function have been demonstrated in β-cell-specific autophagy-deficient mice, which exhibit decreased β-cell mass and insulin release. Furthermore, mitochondrial swelling, together with deficient ATP production, which suggests mitochondrial dysfunction, was observed in β-cells in which critical autophagy genes were deleted^3,4^. Although these results suggest important roles of mitophagy in β-cell function, the roles of authentic mitophagy in β-cell function have not been clearly shown, since mice with β-cell-specific knockout (KO) of *Parkin*, one of the most crucial elements of mitophagy, did not exhibit overt abnormalities in mitochondrial function or morphology or abnormalities in pancreatic β-cell function^5^.

In this review, the roles of mitophagy in the pancreatic β-cell response to metabolic or mitochondrial stress will be discussed, with an emphasis on TFEB, which is a master regulator of lysosomal biogenesis and autophagy gene expression, and lysosomal Ca^2+^, which can activate TFEB after being released into the cytoplasm through lysosomal Ca^2+^ exit channels in response to metabolic or mitochondrial stressors^6^.

## Molecular mechanism underlying bulk autophagy

Bulk autophagy sequesters portions of the cytoplasm in a phagophore that is enclosed by double membranes and targets these portions of the cytoplasm for lysosomal degradation. The bulk autophagic process can be broadly divided into three stages: nucleation, expansion, and degradation.

The UNC51-like kinase 1 (ULK1) complex and Bcl-2-interacting myosin-like coiled-coil protein (BECLIN 1) complex are key players in the nucleation stage. mTORC1 inhibition by nutrient starvation or rapamycin treatment leads to mTORC1 dissociation from the ULK complex, and mTORC1 phosphorylates ATG13 and FIP200 to initiate autophagy^7^. BECLIN 1 forms a complex with VPS34, VPS15, and ATG14L. VPS34, which is a class III phosphatidylinositol-3-kinase (PI3K), produces phosphatidylinositol-3-phosphate (PI3P) and recruits the proteins double FYVE-containing protein 1 (DFCP1), WD Repeat Domain, Phosphoinositide Interacting 2 (WIPI2) as a PI3P-binding effector protein, and ATG to initiate phagophore nucleation.

Autophagosome expansion machinery consists of two ubiquitin-like axes. ATG7 is an E1-like enzyme, and ATG10 and ATG3 are E2-like enzymes. Although there is no E3-like enzyme, the ATG12-ATG5-ATG16L1 complex acts as an E3-like enzyme complex^7^. ATG8 family members, including microtubule-associated protein 1 light chain 3 (LC3), which is a ubiquitin-like protein, are conjugated to Atg3 via the action of ATG7, and the ATG8-ATG3 intermediate is recruited to the isolation membrane via interaction with the ATG12-ATG5-ATG16L1 complex that is bound to the isolation membrane. ATG8 is then conjugated to the lipid target phosphatidylethanolamine (PE), forming LC-II^7^. There are six ATG8 family members (LC3A, LC3B, LC3C, GABARAP, GABARAPL1, and GABARAPL2), and each member may play a nonredundant role^8^.

When autophagosome formation is completed, autophagosomes fuse with lysosomes, and lysosomal enzymes induce the hydrolysis of the sequestered cellular organelles or macromolecules. Thus, lysosomes are the effector organelles of the autophagic process. For more detailed information regarding the molecular and cellular mechanisms underlying autophagy, readers are encouraged to consult excellent recent reviews^9^.

## Machinery of mitophagy

In contrast to bulk autophagy, which nonselectively degrades cytoplasmic constituents, selective autophagy targets specific organelles or molecules for lysosomal degradation. Selective autophagy is essential for the maintenance of the function and integrity of cellular organelles, such as mitochondria, ER, peroxisomes, or lysosomes. There are several types of selective autophagy, and each type of selective autophagy has its own specific machinery in addition to the common autophagic machinery.

One of the most notable examples of selective autophagy is mitophagy. The maintenance of mitochondrial integrity is critical for cell survival and function. Mitochondria are the site of electron transfer and produce abundant ROS radicals due to the leakage of electrons. Mitochondrial DNA is not well protected by nucleosome-like structures due to its partial single-stranded conformation^10^. Hence, the maintenance of mitochondrial function by mitophagy is critical for the maintenance of intracellular redox balance and energy homeostasis.

**Fig. 1:**
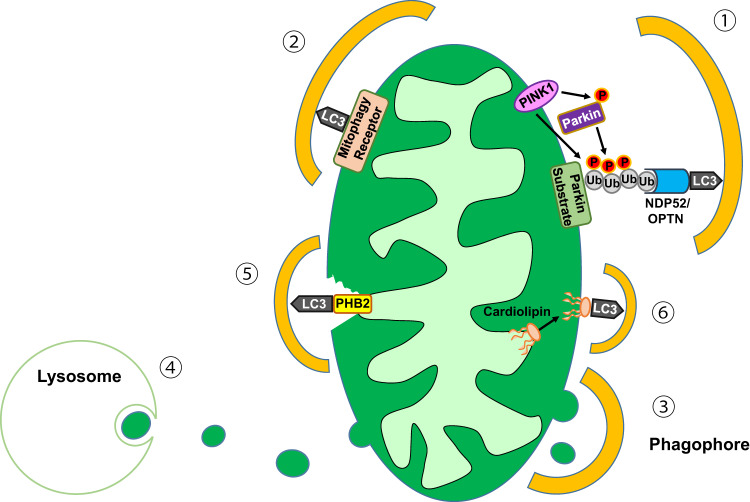
Machinery of mitophagy. (①) During Ub-mediated mitophagy, PINK1 accumulates on the OMM of depolarized mitochondria and induces Ub phosphorylation and PARKIN recruitment. Parkin, which is an E3 ligase, induces the ubiquitination of target proteins, which is recognized by mitophagy receptors such as NDP52 or OPTN and initiates autophagosome formation. (②) During receptor-mediated mitophagy, mitophagy receptors on the OMM that harbor LC3-interacting region (LIR) domains, such as NIX, BNIP3, or FUNDC1, are directly conjugated to ATG8 family members, inducing mitophagy. (③) During piecemeal mitophagy, small parts of mitochondria bud off from the OMM and are surrounded by the LC3C phagophore. (④) During micromitophagy, mitochondria-derived vesicles (MDVs) containing oxidized mitochondrial proteins bud off from the OMM and are directly internalized into multivesicular bodies or lysosomes through invagination without forming double-membrane structures. (⑤) Mitophagy targets on the IMM, such as PHB2, can be recognized by LC3 after the degradation of OMM proteins. (⑥) Cardiolipin on the IMM moves to the OMM during mitochondrial stress and can be recognized by ATG8 family members, such as LC3A.

Mitophagy can be broadly classified into two categories. One category is ubiquitin-mediated mitophagy, and the other category is receptor-mediated mitophagy. During ubiquitin-mediated autophagy, ubiquitin on the surface of stressed mitochondria recruits the autophagy machinery, inducing the autophagic clearance of mitochondria. One of the most well-known and important examples of ubiquitin-mediated mitophagy is PTEN-induced putative kinase 1 (PINK1)-PARKIN-mediated mitophagy. PINK1 is a sensor of mitochondrial depolarization, as PINK1 accumulates on the outer mitochondrial membrane (OMM) of depolarized mitochondria. When the mitochondrial potential is intact, PINK1 is rapidly imported into the mitochondrial intermembrane space through its interaction with mitochondrial translocases of the outer (TOM) and inner mitochondria membranes (TIM23)^11^ and degraded by presenilin-associated rhomboid-like protein (PARL)^12^. PINK1 on the OMM of depolarized mitochondria phosphorylates ubiquitin^13^ and recruits PARKIN, an E3 ligase, which induces the ubiquitination of several OMM proteins, including mitofusin (MFN), PORIN, MIRO, and VDAC. Ubiquitinated PARKIN target molecules recruit autophagy receptors that contain both ubiquitin-binding domain and LC3-interacting region (LIR) domain, such as NDP52 or OPTN, as mitophagy receptors. TBK1, which is an important regulator of innate immune signaling, activates NDP52 and OPTN to facilitate mitophagy^14,15^. Once tethered to the cargos on mitochondria, autophagy (mitophagy) receptors can activate ULK1 together with TBK1 and thereby initiate mitophagy^16^ (Fig. 1).

During receptor-mediated mitophagy, LC3 proteins are directly recruited to mitochondrial target proteins that are localized to the OMM and harbor an LIR domain, such as BNIP3, NIX (also called BNIP3L), FUNDC1, BCL2L13, or FKBP8^17,18^ (Fig. 1). In addition to OMM proteins, the targeting of mitochondrial inner membrane (IMM) proteins has been reported. Prohibitin II (PHB2), which is an IMM protein, can contribute to the clearance of damaged mitochondria via interaction with LC3 after the proteasomal degradation of OMM proteins^19^ (Fig. 1). PHB2-dependent mitophagy has been reported to be required for the clearance of paternal mitochondria after fertilization.

In addition to the two major types of mitophagy, several other types of mitophagy have been described. Piecemeal-type basal and oxidative phosphorylation-induced mitophagy have been described and occur independently of PARKIN^20,21^ (Fig. 1). Micromitophagy is a special form of mitophagy that is characterized by direct invagination of mitochondria into multivesicular bodies (MVBs) or lysosomes without the formation of autophagosomes. Damaged or dysfunctional mitochondria are targeted to the micromitophagy pathway through the budding-off of mitochondria-derived vesicles (MDVs) that are enriched in oxidized mitochondrial proteins, followed by their internalization into MVBs and fusion to lysosomes^22^ (Fig. 1). In addition to LIR domain-containing proteins acting via their interactions with LC3 family members, lipids such as cardiolipin may function as mitophagy receptors^23^. Cardiolipin on the IMM has been reported to move to the OMM via a process called ‘cardiolipin externalization’ during mitochondrial stress, and then, it binds to LC3 family members such as LC3A to initiate mitophagy (Fig. 1).

While the LC3 family is critical for mitophagy, LC3 family members might not be essential for autophagosome formation during mitophagy since autophagosome formation was still observed in hexa KO cells that lacked all 3 *Lc3* and 3 *Gabarap* members^24^. Instead of autophagosome formation, LC3 family members, particularly GABARAP subfamily members, have been shown to be critical for (mito)autophagosome-lysosome fusion^24^. *Atg5*-independent clearance of mitochondria through alternative autophagy has also been reported to occur during erythrocyte maturation^25^.

The role of mitochondrial fission in the execution of mitophagy has been suggested since mitochondrial fission might facilitate mitophagy by dividing whole mitochondria into small fragments that are susceptible to autophagic engulfment. However, DRP1-mediated mitochondrial fission has been shown to protect mitochondria against nonselective mitophagy and the spread of damage by limiting PINK1-PARKIN activity to specific mitochondrial subdomains^26^. Another recent paper reported that DRP1-mediated mitochondrial fission at the mitochondrial periphery leads to the shedding of smaller mitochondrial fragments for mitophagy, while the same DRP1-mediated mitochondrial fission at the mitochondrial mid zone leads to mitochondrial proliferation^27^. Thus, the role of mitochondrial fission in mitophagy might differ depending on the cellular context.

## TFEB in mitophagy

Transcription factor EB (TFEB), which is a member of the Microphthalmia family of bHLH-LZ transcription factors (MiT/TFE), is a master regulator of lysosome biogenesis and autophagy gene expression^28^. Lysosomal Ca^2+^ release has been shown to play roles in TFEB activation during starvation-induced autophagy, leading to the activation of calcineurin, which is one of the most important phosphatases for TFEB dephosphorylation and nuclear translocation^6^. TFEB family members have also been shown to play an important role in mitophagy because TFEB is activated by mitochondrial stressors^29,30^, and *Parkin*-transfected *TFEB/MITF/TFE3*-KO HeLa cells exhibited defective stress-induced mitophagy^29^.

Similar to starvation-induced autophagy, the release of lysosomal Ca^2+^ has been reported to occur during mitochondrial stress-induced mitophagy^30^, and this process is mediated by mitochondrial ROS-induced activation of the TRPML1 lysosomal Ca^2+^ channel^31^. TRPML1 expression on late endosomes and lysosomes is important for autophagosome fusion, mitophagy, and lysosomal adaptation to starvation^32^. The physiological ligand for TRPML1 channel activation is PI(3,5)P2, which is generated by PIKfyve, but ROS can activate TRPML1 channels under nonphysiological or pathological conditions^31^. Subsequently, Ca^2+^ release through the TRPML1 channel can activate TFEB and mitophagy. However, the detailed mechanism underlying lysosomal Ca^2+^ release that allows for the full progression of mitophagy despite the small capacity of lysosomes as Ca^2+^ reservoirs^33^ is unclear. In addition to Ca^2+^-mediated TFEB activation, GABARAP-dependent relocalization of folliculin (FLCN) in complex with FNIP, which is a binding partner for FLCN, on the lysosomal membrane and their sequestration in a process called “CASM” (Conjugation of ATG8 to single membranes) have been reported to contribute to mitophagy^34^. Since FLCN is a GTPase-activating protein (GAP) that targets RagC/D GTPase, which is necessary for mTORC1-mediated TFEB phosphorylation^35^, sequestration of the FLCN/FNIP complex on the lysosomal membrane might inhibit TFEB phosphorylation and activate TFEB. CASM might belong to a large group of phenomena called ‘Atg8ylation’ or Atg8 conjugation to a single membrane, which might play a role in immunity, signal transduction and metabolism^36^. While FLCN is important for mTORC1-induced TFEB phosphorylation, FLCN-mediated RagC/D GTPase activity has been reported to be dispensable for interactions of mTORC1 with other substrates, including S6K or 4E-BP1^35^.

## Autophagy of pancreatic β-cells

Since autophagy controls intracellular nutrient homeostasis and the integrity of organelles that are critical for energy balances, dysregulation of autophagy is likely to contribute to the pathogenesis of metabolic disorders. Hence, changes of autophagy in metabolic tissues have been extensively studied in metabolic diseases^37,38^. Previous studies have reported that autophagy can protect pancreatic β-cells against metabolic stress, such as palmitic acid (PA)-induced lipotoxicity, in vitro^39,40^. Autophagy has also been shown to protect β-cells from deleterious effects resulting from the accumulation of cytotoxic human islet amyloid polypeptide (hIAPP) oligomers that could play an important role in the development of human diabetes characterized by islet amyloid accumulation^41^. Recently, cytoprotective β-cell autophagy was reported to be enhanced by complement C3, which binds to ATG16L1, enhancing autophagic activity^42^.

The role of β-cell autophagy in vivo has also been extensively studied, mostly with genetic models. In pancreatic β-cells in which autophagy genes were deleted, abnormal morphology and function of mitochondria were observed^3,43,44^. Reduced Ca^2+^ transient and defective insulin release after glucose challenge occurred in pancreatic β-cells from these mice, suggesting mitochondrial dysfunction^3,4^. In addition to the role of autophagy in β-cell physiology, dysregulated β-cell autophagy might contribute to the development of diabetes. While β-cell-specific *Atg7*-KO mice showed only mild hyperglycemia, they developed severe diabetes when fed a high-fat diet (HFD)^44^; these results suggested that autophagy is important for β-cell adaptation to metabolic stress. Insufficient autophagy could be an underlying cause of the pathogenesis of diabetes, likely due to compromised adaptation to metabolic stress, reduced lipid clearance or lipophagy, and impaired removal of stressed or dysfunctional mitochondria or ER^45^. Consistently, TFEB activation in pancreatic islets has been reported to be compromised in patients with diabetes, which might indicate reduced autophagic activity^46^.

In addition to classical macroautophagy, other types of autophagy have been reported to occur in pancreatic β-cells, such as starvation-induced nascent granule degradation (SINGD), vesicophagy, microautophagy, or Golgi membrane-associated degradation (GOMED)^47^.

## Mitophagy of pancreatic β-cells

As discussed, the importance of autophagy and its functional role in pancreatic β-cells have been extensively studied, and mitochondrial abnormalities were observed in autophagy-deficient β-cells^3^. The role of the mitophagy receptor FUNDC1 in metabolic syndrome in vivo has also been reported^17^. However, the functional role of bona fide mitophagy in pancreatic β-cells was not clearly demonstrated in a paper that studied *Parkin*-KO mice. While mitochondrial protein turnover after acute mitochondrial stress was delayed by *Parkin* knockdown (KD), β-cell-specific *Parkin*-KO mice did not show abnormal β-cell function or glucose profiles before or after HFD feeding^5^. Islet morphology was also intact in β-cell-specific *Parkin*-KO mice. Such discrepancies could be attributable to compensatory changes in *Parkin*-KO β-cells or PARKIN-independent mitophagy^21,48^. While PARKIN could be important for TFEB activation by mitochondrial stressors^29^, mitophagy that is activated through the TFEB pathway might not entirely depend on PARKIN.

Mice with global *Parkin* KO showed slightly different metabolic features. *Parkin*-KO mice have been reported to have exacerbated glucose intolerance and further impaired insulin release after treatment with streptozotocin and PFT-α, which is an inhibitor of p53, compared to wild-type mice^43^, suggesting a protective role of PARKIN-mediated mitophagy in stressed β-cells. Additionally, global *Parkin*-KO mice showed abnormal lipid metabolism. *Parkin*-KO mice showed resistance to HFD-induced weight gain and adiposity, which was attributed to less pronounced induction of lipid transport proteins such as CD36, Sr-B1, and L-FABP due to *Parkin* KO^49,50^. On the other hand, no specific abnormalities of adipose tissues were observed in adipocyte-specific *Parkin*-KO mice except for a mild increase in fatty acid uptake and β-oxidation of long-chain fatty acids^5^, indicating that complex context-dependent interaction between metabolic tissues influences in vivo phenotypes.

The role of PINK, which is an upstream kinase of Parkin, in β-cell function has also been studied. *Pink1*-KO mice did not exhibit reduced β-cell function or glucose intolerance, although NADH generation and mitochondrial potential after glucose challenge were reduced in *Pink1*-KO β-cells^51^. Basal and glucose-stimulated insulin release were increased in the pancreatic islets of *Pink1*-KO mice, which might be related to high basal cytosolic Ca^2+^ content^51^. However, baseline mitophagy was reportedly higher in the pancreatic islets of *Pink1*-KO mice, which could be an adaptive increase in PINK1-independent mitophagy^52^. Another paper reported that *Pink1*-KO mice exhibited greater weight gain after HFD feeding, which was associated with mitochondrial dysfunction in brown adipose tissue (BAT), conversion of BAT adipocytes to white adipose tissue-like adipocytes and insulin resistance^53^.

Roles of another upstream regulator of PARKIN in β-cell mitophagy have been reported. *Clec16a*, which is a type 1 diabetes susceptibility gene^54^, has been reported to regulate PARKIN expression through its interaction with NRDP1, an E3 ligase^55^. The CLEC16A-NRDP1-USP8 complex might regulate mitophagy by balancing PARKIN ubiquitination and deubiquitination, while disruption of the CLEC16A-NRDP1-USP8 complex by metabolic stressors might induce impaired mitophagy and β-cell dysfunction^56,57^. β-cells with genetic disruption of *Clec16a* were also more susceptible to cytokine-induced death in vitro, which might be related to deficient cytokine-induced mitophagy and consequent mitochondrial dysfunction. Furthermore, β-cell-specific *Clec16a*-KO mice developed more severe diabetes after multiple low-dose streptozotocin treatments a condition mimicking type 1 autoimmune diabetes^58^. *Clec16a*, in turn, has been reported to be regulated by PDX1, which is a well-known homeodomain transcription factor that is involved in β-cell development and diabetes, since *Clec16a* mRNA expression was reduced in *Pdx1*-deficient pancreatic islets^57^. A segment of CLEC16A that is critical for mitophagy has been reported to lie in the C-terminal intrinsically disordered protein region (IDPR) with proline bias^59^. A recent paper reported that *Clec16a* truncation mutations were associated with impaired endosomal trafficking and severe neurodevelopmental disorders, such as microcephaly and growth retardation^60^.

Another potential participant in PARKIN-mediated mitophagy could be MIRO1, which is a PARKIN target^61^ and regulates mitochondrial mobility^62^. *Miro1*-KO β-cells displayed mitochondrial dysfunction and dysregulated insulin release or mitophagy. Furthermore, *Miro1*-KO mice developed an aggravated metabolic profile after HFD feeding^63^. Intriguingly, expression of *Miro1* was reduced in the pancreatic islets of patients or experimental animals with diabetes^63^, which is consistent with a possible role of mitophagy deficiency in the development of diabetes. The role of mitochondrial transcription factor B2 (*Tfb2m*), one of the important transcriptional regulators of mitochondrial genes together with *Tfam*^64^, in the mitophagy of pancreatic β-cells has also been reported. In *Tfb2m*-KO β-cells, autophagosome-lysosome fusion was impaired, leading to reduced mitophagy, mitochondrial dysfunction, and deficient insulin release^65^.

Although most papers that studied mitophagy in pancreatic β-cells suggested beneficial roles of mitophagy in β-cell function, detrimental roles of mitophagy in β-cell function or viability have been reported. Expression of *Nor1*, which belongs to the *Nr4a* family, has been reported to be increased in the pancreatic islets of type 2 diabetes patients, and overexpression of *Nor1* has been reported to induce pancreatic β-cell apoptosis and reduce β-cell mass via increased mitophagy and mitochondrial fragmentation^66^. Another example of the deleterious effect of mitophagy on β-cell function is MitoNEET. Genetic overexpression of *MitoNEET*, which is a mitochondrial outer membrane iron-sulfur protein, induced PARKIN and PARKIN-dependent mitophagy, which led to decreased β-cell mass, impaired insulin release, and glucose intolerance. This phenotype was abrogated by crossing these mice with *Parkin*-KO mice, suggesting a harmful effect of excessive PARKIN-mediated mitophagy on β-cell function^67^.

## Stress-induced mitophagy in pancreatic β-cells

Since mitophagy is critical in the cellular response to mitochondrial stress, stress-induced mitophagy in pancreatic islet cells has been studied. To induce mitochondrial stress, INS-1 insulinoma cells were treated with rotenone, a mitochondrial complex I inhibitor, or antimycin A, a complex III inhibitor, in combination with oligomycin to inhibit the reverse activity of F_1_F_0_-ATPase maintaining mitochondrial potential^68^. Rotenone or the oligomycin/antimycin A (O/A) combination induced substantial mitophagy as assessed by either transfection with *pMito-Keima* encoding a pH-sensitive probe conjugated to a mitochondria-targeting sequence^69^ or by RFP-LC3 colocalization with TOM20, which is an OMM protein^70^.

Since TFEB is activated by mitochondrial stressors and the TFEB family plays a role in mitophagy^29^, the roles of TFEB in β-cell mitophagy were examined. Nuclear translocation of TFEB or TFE3 was observed after the treatment of INS-1 cells with rotenone or O/A. The mitophagy that occurred after treatment with rotenone or O/A was dependent on the TFEB family because *Tfeb* or *Tfe3* KO reduced mitophagy after treatment with rotenone or O/A. Activation or nuclear translocation of TFEB family members, such as TFEB or TFE3, by mitochondrial stressors appeared to occur due to ROS that were generated by mitochondrial stressors, since *N*-acetyl cysteine (NAC), which is an antioxidant, inhibited TFEB activation. Specifically, the accumulation of mitochondrial superoxide, as shown by MitoSOX staining, was observed after treatment with rotenone or O/A. Furthermore, abrogation of mitochondrial ROS by MitoTEMPO, which is a scavenger of mitochondrial ROS, inhibited TFEB activation, demonstrating the specific roles of mitochondrial ROS in TFEB activation^70^.

In addition to the effects of mitochondrial stressors, the effects of metabolic stress on mitophagy have also been studied via the administration of PA, which is an in vitro effector of metabolic stress^71–73^. PA was able to induce mitochondrial ROS, as determined by MitoSOX staining, at 4 h after treatment, which could occur due to mitochondrial electron transport chain inhibition^74^. PA also induced TFEB nuclear translocation, which progressively increased between 4–16 h after PA treatment. TFEB activation after PA treatment was inhibited by NAC or MitoTEMPO, suggesting that TFEB is activated as a response to ROS production due to PA-induced mitochondrial stress. Mitophagy was also induced by PA, probably due to TFEB activation^70^.

**Fig. 2:**
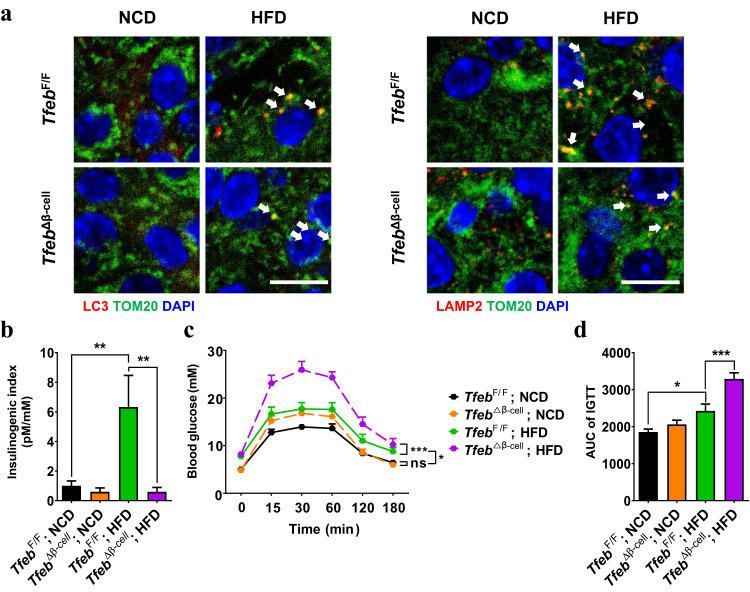
Protective effect of stress-induced TFEB-dependent mitophagy against metabolic stress. **a** Mitophagy was detected by the colocalization of LC3 puncta with TOM20, which is an OMM protein, (left) or the colocalization of LAMP2, which is a lysosomal membrane protein, with TOM20 (right) in pancreatic islets of *Tfeb*^∆β-cell^ and control *Tfeb*^F/F^ mice fed a normal chow diet (NCD) or high-fat diet (HFD) for 12 weeks (arrows). Insulin immunofluorescence is not shown for clarity. Mitophagy in β-cells of HFD-fed mice was reduced by *Tfeb* KO. (scale bar, 10 μm) **b** Insulinogenic index of *Tfeb*^∆β-cell^ and *Tfeb*^F/F^ mice fed an HFD for 12 weeks, showing more severe β-cell failure in *Tfeb*^∆β-cell^ mice than in *Tfeb*^F/F^ mice. (mean ± SEM, *n* = 9 each) **c** Glucose tolerance test in *Tfeb*^∆β-cell^ and *Tfeb*^F/F^ mice fed an NCD or HFD for 12 weeks. (mean ± SEM, *n* = 14 each) **d** Area under the curve (AUC) of the curve in (**c**) shows a further exacerbated metabolic profile in *Tfeb*^∆β-cell^ mice fed an HFD compared to control *Tfeb*^F/F^ mice fed an HFD. Reproduced from “Park et al. Lysosomal Ca^2+^-mediated TFEB activation modulate mitophagy and functional adaptation of pancreatic β-cells to metabolic stress. Nat Commun 2022;13:1300”, with modifications.

Metabolic stress also activated mitophagy in pancreatic islets in vivo. In vivo mitophagy, as determined by RFP-LC3 colocalization with TOM20 or by LAMP2 colocalization with TOM20^75^, was significantly increased in pancreatic islets when mice were fed an HFD for 8 weeks (Fig. 2a), indicating that mitophagy was activated by metabolic stress in vivo. An increase in mitophagy after HFD feeding was also observed in a mouse model that expressed conditional mitochondrial matrix targeting mitophagy receptor (CMMR), which is a mitophagy reporter, in pancreatic β-cells^76^. Increased mitophagy after HFD feeding could be an adaptive response of β-cells to metabolic stress-induced mitochondrial stress in vivo. Indeed, ROS accumulation, as detected by 4-hydroxynonenal (HNE) staining, was observed in the islets of HFD-fed mice. ROS accumulation in the pancreatic islets of HFD-fed mice initiates diverse stress responses, including TFEB activation and mitophagy. These results are similar to the increased mitophagy that was observed in cardiomyocytes of HFD-fed mice^77^, while decreased mitophagy in liver tissues after HFD feeding has also been reported^78^. Such discrepancies could occur due to different durations of metabolic stress or different methods of mitophagy detection. These results suggest that the mitophagic response to metabolic stress could be different depending on the tissue or cellular context.

## Role of Ca^2+^ in stress-induced TFEB activation and mitophagy in β-cells

**Fig. 3:**
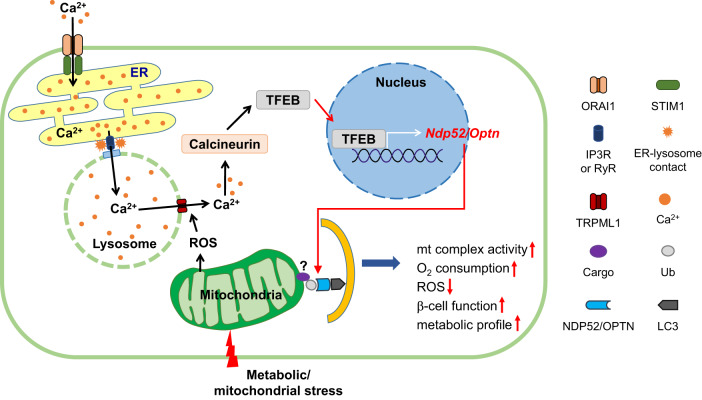
A schematic illustration of the mechanism underlying the induction of mitophagy in pancreatic β-cells by mitochondrial or metabolic stress. Mitochondrial or metabolic stress induces the generation of mitochondrial reactive oxygen species (ROS), which activate lysosomal Ca^2+^ exit channels, such as the TRPML1 channel, and consequently increase cytosolic [Ca^2+^]. TFEB is activated through calcineurin-mediated dephosphorylation and then moves to the nucleus to induce the expression of target genes, including mitophagy receptor genes such as *Ndp52* or *Optn*. The induction of mitophagy receptors facilitates mitophagy through interaction with LC3 and mitophagy cargo, but the nature of the cargo is unclear. Lysosomal Ca^2+^ release is replenished by Ca^2+^from the ER, which is the largest intracellular Ca^2+^ reservoir, and this is facilitated by ER-lysosome contact (ER**→**lysosome Ca^2+^ refilling). ER Ca^2+^ depletion, in turn, activates store-operated Ca^2+^ entry (SOCE) from extracellular Ca^2+^. Mitophagy induction by mitochondrial or metabolic stress helps maintain the mitochondrial function and insulin release of pancreatic β-cells. (mt, mitochondrial).

As a mechanism underlying mitophagy in pancreatic β-cells in response to mitochondrial stressors, the roles of mitochondrial stress-activated TFEB have been investigated. Since TFEB has been reported to be activated by Ca^2+^-mediated calcineurin under conditions of starvation^6^ or mitochondrial stressor treatment^29,31^, the roles of calcineurin in the mitophagy of insulinoma cells have been examined. Indeed, TFEB nuclear translocation due to mitochondrial stressors was abrogated by transfection with a dominant-negative (DN) mutant^79^, suggesting a role of calcineurin in mitochondrial stress-induced TFEB activation (Fig. 3). Calcineurin is activated by increased cytosolic Ca^2+^ levels since BAPTA-AM, which is a chelator of intracellular Ca^2+^, abrogated the TFEB activation induced by mitochondrial stressors^70^. The increase in cytosolic Ca^2+^ by mitochondrial stressors likely occurs due to mitochondrial ROS because quenchers of mitochondrial ROS inhibited the increase in cytosolic Ca^2+^ after mitochondrial stressor treatment (Fig. 3). Consistent with the role of calcineurin in TFEB activation, TFEB was dephosphorylated by mitochondrial stressor treatment in vitro^70^, likely through calcineurin activation induced by the mitochondrial ROS-induced increase in intracellular Ca^2+^. Dephosphorylation of TFEB liberates TFEB from its interaction with the 14-3-3 protein, leading to nuclear translocation. Similar to these results, the effects of a glucagon-like peptide-1 (GLP-1) receptor agonist on protecting β-cells against glucolipotoxicity have also been ascribed to the activation of autophagy through the EPAC/Ca^2+^/calcineurin pathway^80^. The role of calcineurin in mediating TFEB activation via Ca^2+^ that is released from lysosomes and mitophagy induction might be related to the well-known adverse effects of calcineurin inhibitors, such as cyclosporine A or FK506, which can induce β-cell dysfunction and posttransplantation diabetes mellitus (PTDM)^81,82^.

As a source of Ca^2+^, the role of lysosomal Ca^2+^ in the activation of calcineurin and TFEB after exposure to mitochondrial stressors has been studied, since lysosomal Ca^2+^ release is important for the activation of calcineurin and TFEB during starvation-induced autophagy^6^, and mitochondrial ROS can induce lysosomal Ca^2+^ release by activating lysosomal Ca^2+^ exit channels^31^. Indeed, lysosomal Ca^2+^ content ([Ca^2+^]_Lys_) was decreased by mitochondrial stressors in vitro, which is consistent with the release of lysosomal Ca^2+^. Lysosomal Ca^2+^ release is likely the cause of the increased cytosolic Ca^2+^ contents observed in INS-1 cells after exposure to mitochondrial stressors or metabolic stressors^70^ (Fig. 3). The lysosomal Ca^2+^ channel that is responsible for Ca^2+^ release after exposure to mitochondrial stress appears to be TRPML1, since mitochondrial stress-induced lysosomal Ca^2+^ release was attenuated by ML-SI3, an inhibitor of the TRPML1 channel^83^, or KD of TRPML1^70^, which is consistent with the role of TRPML1 in autophagy and lysosomal function^32^. The TRPM1 channel can be activated by mitochondrial ROS in addition to its physiological ligand, PI(3,5)P_2_^30^. In addition to mitochondrial stressors, PA can also reduce [Ca^2+^]_Lys_ and increase cytosolic Ca^2+^ contents in INS-1 cells^70^, which can explain TFEB activation and mitophagy induction by PA^70^; these results suggest a similar mechanism of mitophagy induction by mitochondrial and metabolic stressors.

Although these results suggest that lysosomal Ca^2+^ release in response to mitochondrial or metabolic stress can activate TFEB and mitophagy, lysosomal Ca^2+^ might not be sufficient to guarantee the full progression of Ca^2+^-dependent intracellular events because of the small lysosomal volume^33^. [Ca^2+^]_Lys_ is comparable to that in the ER ([Ca^2+^]_ER_); however, the volume of lysosomes is 1/10 of the volume of the ER, which is the largest intracellular Ca^2+^ reservoir, and thus, replenishment of the deficient lysosomal Ca^2+^ reservoir from the ER might occur after lysosomal Ca^2+^ depletion to sustain lysosomal Ca^2+^ release^84^. When the possible occurrence of ER→lysosome Ca^2+^ flux after lysosomal Ca^2+^ depletion was studied, a decrease in [Ca^2+^]_ER_ was observed after treatment with mitochondrial stressors if extracellular Ca^2+^ was removed to inhibit store-operated Ca^2+^ entry (SOCE). Furthermore, when [Ca^2+^]_ER_ and [Ca^2+^]_Lys_ were simultaneously traced, a decrease in [Ca^2+^]_ER_ occurred in parallel with the recovery of [Ca^2+^]_Lys_ after the removal of mitochondrial stressors^70^, strongly suggesting the occurrence of ER→lysosome Ca^2+^ refilling during mitochondrial stressor-induced mitophagy (Fig. 3). The ER Ca^2+^ exit channels that are responsible for ER→lysosome Ca^2+^ refilling during mitophagy appear to be the IP3 receptor (IP3R) channel and ryanodine receptor (RyR) channel because both xestospongin C, which is an inhibitor of the IP3R channel, and dantrolene, which is an inhibitor of the RyR channel, inhibited the recovery of [Ca^2+^]_Lys_ after the removal of mitochondrial stressors. Xestospongin C and dantrolene also significantly reduced mitophagy after exposure to mitochondrial stressors, probably due to inhibition of ER→lysosome Ca^2+^ refilling of the diminished lysosomal Ca^2+^ stores^70^ (Fig. 3). ER Ca^2+^ release through the IP3R channel has also been suggested to contribute to TBK1 activation or NDP52 recruitment to depolarized mitochondria, likely via TBC1D9, which is a Rab GTPase-activating protein (RabGAP), and this could lead to mitophagy or xenophagy^85^.

As a potential mechanism underlying Ca^2+^ flux between organelles, contact between the ER and lysosome has been studied because organelle contact could facilitate the transport of ions, such as Ca^2+^, between these organelles. Although ER-mitochondria contact has been extensively characterized, ER-lysosome contact has also been reported after lysosomal membrane permeabilization induces lysosomal Ca^2+^ release^86^. Indeed, in insulinoma cells exposed to mitochondrial stressors, ER-lysosome contact was clearly visualized by proximity ligation assay (PLA) and pBirA biotin ligase complementation assay^70^. Such physical contact between the ER and lysosomes might help facilitate the ER→lysosome Ca^2+^ flux (Fig. 3). As ER Ca^2+^ content decreases after exposure to mitochondrial stressors, probably due to ER→lysosome Ca^2+^ refilling, SOCE is likely to be activated. Indeed, abrogation of SOCE by BTP2 or extracellular Ca^2+^ chelation using EGTA significantly exacerbated ER Ca^2+^ depletion and reduced mitophagy in response to mitochondrial stressors. Aggregation of STIM1, which is a Ca^2+^ sensor in the ER, and its colocalization with ORAI1, which is a SOCE channel on the plasma membrane, were also observed after treatment with mitochondrial stressors, which could facilitate ORAI1-mediated extracellular Ca^2+^ influx in response to mitochondrial stressors. This finding could be additional evidence to support the occurrence of ER Ca^2+^ depletion after exposure to mitochondrial or metabolic stressors, likely due to lysosomal Ca^2+^ release and ER→lysosome Ca^2+^ refilling^87^. These results suggest a crucial role of Ca^2+^ release from lysosomes in conjunction with ER→lysosome Ca^2+^ refilling during TFEB activation and progression of mitophagy in response to mitochondrial or metabolic stressors (Fig. 3).

In addition to Ca^2+^ release, other mechanisms might contribute to stress-induced TFEB activation, such as ATG8 conjugation to a single membrane or DUSP1 induction followed by ERK2 dephosphorylation and TFEB activation^34,88^.

## Functional role of TFEB-mediated mitophagy under conditions of metabolic stress

Although mitophagy was expected to be important in the maintenance of β-cell function and integrity, the role of mitophagy in pancreatic β-cells has been unclear since *Parkin*-KO mice do not exhibit apparent metabolic abnormalities, as discussed^5^. Thus, the functional role of TFEB-mediated mitophagy in β-cells has been studied employing β-cell-specific *Tfeb* KO (*Tfeb*^Δβ-cell^) mice. As discussed, mitophagy was increased by HFD feeding, which appears to be an adaptive change in response to metabolic stress^70^, similar to the findings in other tissues, such as cardiac tissues of HFD-fed mice^77^. In addition to mitophagy, the mitochondrial complex activity of pancreatic islets was also increased by HFD feeding, as assessed by cytochrome *c* oxidase (COX) staining to measure COX activity in vivo^70^. Mitochondrial oxygen consumption, as determined by Seahorse respirometry, was also increased in pancreatic islets of HFD-fed mice. These results are also consistent with enhanced mitochondrial respiration or mitochondrial complex activity in cardiac or liver tissues of HFD-fed mice^89,90^. These mitochondrial changes could also be an adaptation to metabolic stress to meet the metabolic demand caused by obesity and insulin resistance. Such adaptive increases in mitophagy, mitochondrial complex activity, and mitochondrial oxygen consumption in pancreatic islets after HFD feeding appeared to be dependent on TFEB since such changes in mitochondrial indices, including mitophagy, were significantly decreased by β-cell-specific *Tfeb* KO (Fig. 2a). ROS accumulation in pancreatic islets of HFD-fed *Tfeb*^Δβ-cell^ mice was aggravated likely due to the abrogation of adaptive mitochondrial changes by *Tfeb* KO, suggesting that TFEB is important for mediating adaptive changes in mitochondria and mitophagy under conditions of metabolic stress^70^.

Mitophagy is functionally important in the cellular response against mitochondrial stress. Hence, the functional significance of enhanced mitophagy and mitochondrial function under conditions of metabolic stress has been examined by studying the metabolic profile and β-cell function of *Tfeb*^Δβ-cell^ mice. An HFD-induced increase in the insulinogenic index, indicating β-cell adaptation, was observed in wild-type mice but not in *Tfeb*^Δβ-cell^ mice^70^, suggesting that mitophagy could be important in the functional adaptation of β-cells to metabolic stress caused by HFD (Fig. 2b). HFD-fed *Tfeb*^Δβ-cell^ mice displayed further aggravated glucose intolerance compared to HFD-fed control mice likely due to the abrogation of the HFD-induced increase in the insulinogenic index (Fig. 2c, d). These results suggest roles of TFEB-mediated mitophagy in the adaptation to metabolic stress, while roles of other types of selective autophagy or global autophagy that can be modulated by TFEB in the β-cell functional adaptation to metabolic stress cannot be disregarded. Although the TFEB-dependent mitophagy observed in pancreatic islets of HFD-fed mice could be an adaptive response to metabolic stress, without which more severe metabolic dysfunction might occur, such a response might not be sufficient for the full compensation of β-cell dysfunction under conditions of metabolic stress. Consistently, the abnormal accumulation of dysfunctional mitochondria was observed in pancreatic islets of HFD-fed mice despite the functionality of lysosomes, suggesting the insufficient capacity of β-cells to degrade dysfunctional mitochondria via mitophagy under conditions of metabolic stress^76^. Regarding other types of autophagy that could be affected by TFEB activation, the flux of autophagy or ER-phagy was not increased by HFD feeding despite TFEB activation in pancreatic islets^70^. These results are consistent with previous papers reporting no increase in autophagy or ER-phagy flux by PA or HFD feeding^77,91,92^ and could be attributable to distinct molecular machinery that is required for the autophagic removal of different organelles.

TFEB target genes include lysosomal genes, autophagy genes, and mitochondrial genes. When RNA expression was studied to identify TFEB target genes associated with β-cell responses to mitochondrial or metabolic stress, the expression of *Tfeb*, *Tfe3*, and their downstream genes, such as lysosomal genes or autophagy genes, was increased in the pancreatic islets of HFD-fed mice. The expression of mitochondrial genes, including *Tfam*, which is a master regulator of mitochondrial gene expression and mitochondrial biogenesis^64^, was also increased in the pancreatic islets of HFD-fed mice^70^, which is similar to the increased expression of mitochondrial genes in the skeletal muscle of mice that genetically overexpressed *Tfeb*^93^. Induction of mitochondrial gene expression or mitochondrial biogenesis might contribute to the enhanced mitochondrial respiration or mitochondrial complex activity that is observed after HFD feeding, in addition to enhanced mitophagy. Coupling between mitophagy and mitochondrial biogenesis might help maintain mitochondrial homeostasis and mass under stress conditions, despite enhanced mitophagy potentially reducing mitochondrial mass. These changes in the increased expression of lysosomal genes, autophagy genes, and mitochondrial genes in the pancreatic islets of HFD-fed mice were all suppressed in β-cell-specific *Tfeb*-KO mice, suggesting roles for *Tfeb* and its downstream genes in the adaptative response to HFD-mediated metabolic stress. In contrast, reduced *Tfeb* expression in the pancreatic islets of HFD-fed mice has been reported^94^, which could be due to different protocols or durations of HFD feeding and dissimilar methods of measuring gene expression.

During the increase in mitophagy by mitochondrial stressors, the induction of mitophagy receptors could be important since they can initiate mitophagy through mechanisms that include ULK1 recruitment^16^. Indeed, autophagy-related genes induced by metabolic stress in pancreatic islet cells from HFD-fed mice included *Ndp52* and *Optn*, which are well-known mitophagy receptors, and this increased expression was attenuated by β-cell-specific *Tfeb* KO^70^. The expression of other putative mitophagy receptors, such as *Nbr1*, *Tbk1* or *Taxbp1*, was also increased; however, statistically significant differences were not observed. Because NDP52 and OPTN are primary receptors for PARKIN-mediated mitophagy^95^, the *Tfeb*-dependent induction of *Ndp52* and *Optn* in pancreatic islet cells would contribute to the increased mitophagy due to TFEB activation by metabolic or mitochondrial stressors. Induction of *Ndp52* and *Optn* by metabolic stress or mitochondrial stress together with their downregulation by *Tfeb* KO occurred due to the TFEB-mediated transactivation of *Ndp52* and *Optn*, as revealed by chromatin immunoprecipitation (ChIP) assay^70^. Putative TFEB-binding sites containing the Coordinated Lysosomal Expression and Regulation (“CLEAR”) sequence (CACGTG) were found in the promoter regions of the human *NDP52* and *OPTN* genes^96^. The binding of TFEB to the “CLEAR” sites of the *NDP52* and *OPTN* promoters as assessed by ChIP assay was significantly increased by mitochondrial stressors, showing that TFEB binds to the promoters of *NDP52* and *OPTN* under stress conditions^70^. These results suggest that the TFEB-dependent transactivation of *Ndp52* and *Optn* might play a role in stress-induced mitophagy, in addition to the activation of mitophagy receptors by phosphorylation^15^. Consistently, KD of *Ndp52* or *Optn* reduced the induction of mitophagy by mitochondrial stressors^70^. The importance of the induction of mitophagy receptors, such as *Ndp52* or *Optn*, in the execution of mitophagy, was further demonstrated in a recent paper reporting that the recruitment of NDP52 to mitochondria alone could induce mitophagy^16^. Intriguingly, a recent paper employing genome-wide CRISPR screening identified *Ndp52* as a gene that regulates β-cell function and influences the risk of diabetes^97^. In addition to *Ndp52* and *Optn*, the induction of BNIP3 and NIX might contribute to the increased mitophagy in pancreatic β-cells after HFD feeding. The expression of BNIP3 and NIX was increased in a HIF-1α-dependent manner in enlarged pancreatic islets of HFD-fed mice^76^. Furthermore, overexpression and KD of *Bnip3* alone could increase and decrease mitophagy of β-cells, respectively, suggesting that expression of the mitophagy receptor on its own might modulate mitophagy.

These results suggest that TFEB is activated by lysosomal Ca^2+^ release coupled with ER→lysosome Ca^2+^ refilling and that TFEB-dependent induction of mitophagy, probably through the transactivation of mitophagy receptor genes such as *Ndp52* or *Optn*, plays an important role in β-cell adaptation to metabolic stress through enhanced or protected mitochondrial function (Fig. 3).

## Impaired β-cell mitophagy as a cause of pancreatic β-cell dysfunction induced by calcineurin inhibitor

As discussed, mitophagy plays a crucial role in the functional adaptation of pancreatic β-cells to metabolic stress, and *Tfeb* is important in this process of adaptive mitophagy^70^. Additionally, calcineurin is one of the most important phosphatases that induces the dephosphorylation and nuclear translocation/activation of TFEB^6,29,31^, while the roles of other phosphatases, such as protein phosphatase 2A (PP2A), in TFEB dephosphorylation cannot be neglected^98^. Thus, calcineurin inhibitors might be able to inhibit TFEB activation, which might lead to impaired bulk autophagy or mitophagy in pancreatic β-cells. The possibility that TFEB activation could be compromised by calcineurin inhibitors may have clinical implications since calcineurin inhibitors are widely used as immunosuppressive agents in patients with graft rejection^81,99^ or autoimmune diseases^100^. Major adverse effects of calcineurin inhibitors include hyperglycemia or posttransplantation diabetes mellitus (PTDM), which has been attributed to pancreatic β-cell dysfunction and defective insulin release^82^, while other mechanisms such as insulin resistance might also contribute^101^. β-cell dysfunction due to calcineurin inhibitors has been ascribed to impaired FK506-binding protein-cyclic ADP ribose interaction, incomplete closure of ATP-sensitive K^+^ channel or reduced insulin gene transcription^102,103^. However, as TFEB activation by calcineurin is an important mechanism underlying adaptive mitophagy in response to mitochondrial or metabolic stress^70^, the possible inhibition of mitophagy by calcineurin inhibitors might also contribute to the development of PTDM, in addition to other previously known mechanisms underlying β-cell failure induced by calcineurin inhibitors.

**Fig. 4:**
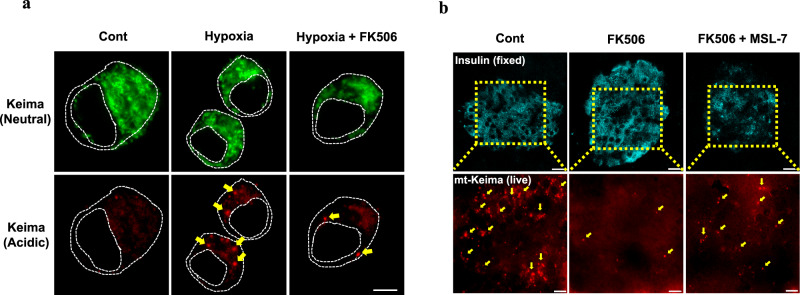
Reduction of mitophagy by FK506, a calcineurin inhibitor. **a** Acidic Mito-Keima red puncta, representing mitophagy (arrows), in INS-1 insulinoma cells transfected with *pMito-Keima* and then incubated in a hypoxic chamber (1% O_2_) for 24 h with or without FK506 were visualized by fluorescence microscopy. Hypoxia-induced mitophagy was suppressed by FK506, which is a calcineurin inhibitor. (Cont, control) (scale bar, 10 μm) **b** Mito-Keima red puncta in live pancreatic tissue (arrows) from *mito-Keima-*transgenic mice after the administration of FK506 in vivo with or without MSL-7, which is an autophagy enhancer that activates TFEB (lower). Islet β-cells were identified by immunofluorescence staining for insulin in adjacent pancreatic sections after fixation (upper). The number of Mito-Keima red puncta in pancreatic islets was decreased by FK506, and this effect was partially restored by the concomitant administration of MSL-7. Cells in the rectangles were magnified. (scale bar, 10 μm).

Indeed, FK506, which is one of the most widely used calcineurin inhibitors^81^, was found to inhibit the induction of mitophagy by mitochondrial stressors or hypoxia, which is a well-known inducer of mitophagy^104^ (Fig. 4a). The recovery of mitochondrial potential and clearance of mitochondrial ROS following the removal of mitochondrial stressors after treatment were delayed by FK506, likely due to impaired mitophagy. Mitochondrial O_2_ consumption and glucose-stimulated insulin release in vitro were also decreased by FK506, likely due to mitochondrial dysfunction^104^. Furthermore, the insulinogenic index, which represents a β-cell function, was suppressed by the in vivo administration of FK506. Mitochondrial complex activity, as determined by COX staining, was also suppressed in the pancreatic islets of mice treated with FK506. In addition, mitophagy in pancreatic islets was also downregulated by FK506 administration in vivo^104^, as evidenced by reduced colocalization of RFP-LC3 puncta with TOM20 or a decreased number of Keima puncta in the pancreatic islets of transgenic mice expressing *mito-Keima*^78^ (Fig. 4b). Based on these results suggesting that calcineurin inhibitors impair TFEB activation and mitophagy, the effects of autophagy enhancers that can activate TFEB on FK506-induced β-cell dysfunction have been studied. MSL-7, which can activate TFEB through calcineurin^105^, was able to restore the mitophagy that was impaired by 100 ng/ml FK506 (a concentration that inhibits TFEB or mitophagy in vitro^104^ and is higher than the concentration in patients treated with FK506)^106^ (Fig. 4b). Furthermore, pancreatic β-cell dysfunction in vivo after FK506 administration was significantly recovered by combined treatment with MSL-7, which was associated with a reversal of FK506-mediated mitophagy suppression^104^. Thus, one mechanism underlying glucose intolerance or diabetes due to calcineurin inhibitors might entail the suppression of mitophagy. These results suggest the role of TFEB-mediated mitophagy in pancreatic β-cell function and suggest the therapeutic potential of inducers of autophagy or mitophagy in the treatment of PTDM.

## Conclusion and perspective

Mitophagy plays a critical role in maintaining the mitochondrial function and the insulin release capability of β-cells. The modulation of mitophagy or autophagy might have therapeutic relevance in the treatment of diabetes accompanied by compromised insulin release or β-cell function associated with metabolic stress. TFEB could be a target for the modulation of autophagy or lysosomal function in metabolic tissues, including β-cells. Lysosomal Ca^2+^ release channels or ER→lysosomal Ca^2+^ flux might also provide novel targets for therapeutic strategies, and this might have implications for the treatment of diverse conditions or diseases associated with changes in lysosomal Ca^2+^ concentrations, such as autophagy, inflammation, or vaccination^70,84^. Glucose intolerance or diabetes associated with the administration of calcineurin inhibitors could be examples that demonstrate the crucial role of mitophagy in the functional maintenance and adaptation of β-cells.

While the overall role of mitophagy in β-cell function was recently demonstrated in a couple of papers^57,70,76^, many questions remain to be addressed. It is unclear how TFEB activation in β-cells due to mitochondrial or metabolic stress leads to mitophagy without inducing other types of selective autophagy or bulk autophagy. Some auxiliary machinery for mitophagy might be specifically induced by mitochondrial or metabolic stress, and the induction of *Ndp52*, *Optn*, *Bnip3*, or *Nix* might be one such mechanism. It is also unclear whether diverse types of mitophagy, such as piecemeal-type mitophagy, micromitophagy, or various receptor-mediated mitophagy, occur in β-cells under basal or stress conditions, and this information might be able to explain the absence of morphological or functional changes in the mitochondria of *Parkin*-KO β-cells. In addition, execution of mitophagy in β-cells to the stage of lysosomal degradation of mitochondrial proteins might need to be confirmed, as dissociation between mitophagy detection using pH-sensitive fluorescent markers and target substrate protein degradation has been reported^107^. The role of lysosomal dysfunction in mitophagy in stressed β-cells needs to be investigated as well, since effectors of metabolic stress, such as PA, induce not only mitochondrial stress but also lysosomal stress, and lysosomal stress impedes the activity or progression of mitophagy or other types of autophagy, including bulk autophagy. For clinical purposes, pharmacological methods that enhance mitophagy without causing mitochondrial stress would be helpful for protection of pancreatic β-cells from mitochondrial or metabolic stress.
